# Hyperhomocysteinemia and intracranial aneurysm: A mendelian randomization study

**DOI:** 10.3389/fneur.2022.948989

**Published:** 2022-09-28

**Authors:** Chencheng Ma, Weiwei Zhang, Lei Mao, Guangjian Zhang, Yuqi Shen, Hanxiao Chang, Xiupeng Xu, Zheng Li, Hua Lu

**Affiliations:** ^1^Department of Neurosurgery, The First Affiliated Hospital of Nanjing Medical University, Nanjing, China; ^2^Department of Neurosurgery, Jiangsu Province Hospital, Nanjing, China; ^3^Department of Ophthalmology, Third Medical Center of Chinese PLA General Hospital, Nanjing, China

**Keywords:** Mendelian randomization, intracranial aneurysm, hyperhomocysteinemia, causality, cerebrovascular disease

## Abstract

**Objective:**

To investigate the link between genetic variants associated with plasma homocysteine levels and risk of intracranial aneurysm (IA) using two-sample Mendelian randomization.

**Methods:**

We used single-nucleotide polymorphisms associated with human plasma homocysteine levels as instrumental variables for the primary analysis in a genome-wide association study of 44,147 subjects of European ancestry. Summary-level statistics were obtained for 79,429 individuals, including 7,495 IA cases and 71,934 controls. To enhance validity, five different Mendelian randomization methods (MR-Egger, weighted median, inverse variance weighted, simple mode, and weighted mode) were used for the analyses.

**Results:**

The inverse variance weighted analysis method produced *P*-values of 0.398 for aneurysmal subarachnoid hemorrhage [odds ratio (OR): 1.104; 95% confidence interval (CI): 0.878–1.387], 0.246 for IA (OR: 1.124; 95% CI: 0.923–1.368), and 0.644 for unruptured IA (OR: 1.126; 95% CI: 0.682–1.858). The MR-Egger analysis showed no association between IAs and homocysteine, with all *P* > 0.05.

**Conclusion:**

Using gene-related instrumental variables, the Mendelian randomization analyses demonstrated a lack of an association between plasma homocysteine levels and IAs or aneurysmal subarachnoid hemorrhage.

## Introduction

Intracranial aneurysm (IA) is confined, pathological dilatations of the walls of intracranial arteries that are at risk of rupture. About 85% of spontaneous subarachnoid hemorrhage (SAH) is due to ruptured IA (1). The incidence of IA was reported to be about 3.2% in a worldwide study with a mean age of 50 years (2). Aneurysmal SAH (aSAH) often has a poor prognosis, with high disability and mortality rates (3, 4). However, the etiopathology of IAs remains unclear.

Hyperhomocysteinemia has been widely reported to be associated with the development of cerebrovascular disease (5–8). Excessive homocysteine levels lead to inflammation of the vessel wall, atherosclerotic plaque formation, endothelial cell damage, smooth muscle cell proliferation, and altered oxidative stress response (9–11). These pathological changes play a critical role in the formation and rupture of IAs (12, 13). We, therefore, speculated that the formation and rupture of IAs may be associated with homocysteine.

Recent studies have shown an association between IAs and hyperhomocysteinemia in the Chinese Han population (14, 15). In 2011, a study reported that hyperhomocysteinemia in a rat model accelerated IA formation (16). However, it has been reported that homocysteine is not associated with the IAs in other races (17). Therefore, the association between IA formation and homocysteine remains unresolved.

Mendelian randomization (MR) is the use of genetic variation in non-experimental data to estimate the causal link between exposure and outcome, and it can reduce the impact of behavioral, social, psychological, and other factors (18). And in recent years, many MR studies have emerged to provide clinical evidence (19–21). This proves that MR is a reliable research method to solve some problems. Using recently published summary data for plasma homocysteine levels and summary data for IA in a genome-wide association study (GWAS), we aimed to analyze the causal connection between homocysteine and IA using two-sample MR.

## Materials and methods

### Genetic instruments and data sources

We used single-nucleotide polymorphisms (SNPs) associated with human plasma homocysteine levels as instrumental variables (IVs) for the primary analysis in a GWAS of 44,147 subjects of European ancestry (22).

We extracted SNPs associated with IA from a large GWAS involving 7,495 IA cases and 71,934 controls (23). The MR analysis was performed on three summary datasets from this GWAS. The three pooled datasets were GWAS of IA (ruptured, unruptured, and unknown rupture status) (*n* = 7,495), UIA-only (*n* = 2,070), and aSAH-only (*n* = 5,140) vs. controls (*n* = 71,934) in individuals of European ancestry.

The following steps were applied to select the best IVs to guarantee the accuracy and validity of the inferences on the causal relationship between the risk of IA and plasma homocysteine. The first step was to select SNPs with thresholds of significant association with the plasma homocysteine levels as IVs. A set of genome-wide statistically significant (*P* < 5 × 10^−8^) SNPs were used as IVs. Second, linkage disequilibrium (LD) must not exist between the selected IVs, because it can lead to interpretation bias. Among the selected SNPs, we performed a clumping step (clumping distance = 10,000 kb, *R*^2^ < 0.001) to reduce the LD during our MR analysis. Third, guaranteeing that the impact of SNPs on outcome and exposure is related to only one allele during MR analysis is an important condition, and in accordance, SNPs with a palindromic structure were removed.

### Standard protocol approval, registration, and patient consent

All the data used in this MR analysis were based on summary data publicly available from the GWASs. Ethical approval and participant consent were not needed as they were previously obtained for each of the original GWASs.

### The assumptions of MR

To investigate the causal impact of the plasma homocysteine on IA, genetic variation was used as an IV in MR. To serve as an IV, the following criteria must be met: the variation must be related to the plasma homocysteine; it must not be related to any confounding factor related to the plasma homocysteine or IA; it must not affect the outcome, except possibly through association with exposure (24). The *F*-statistic, whose formula is *F* = $\frac{R^{2}\;\left(n-k-1\right)}{k\;\left(1-R^{2}\right)}$, is commonly used to evaluate the strength of the correlation between exposure and IVs. Here, *n* represents the number of samples in the GWAS related to exposure, *k* represents the number of IVs, and *R*^2^ is the extent to which IVs explain exposure. When the *F*-statistic is < 10, we usually consider the IVs as weak, which may bias the results somewhat.

### Statistical analysis

We used the inverse variance weighted (IVW), MR-Egger, weighted median, simple mode, and weighted mode methods to evaluate the causal link between IAs and plasma homocysteine. The IVW method is characterized by an analysis that does not take into account the presence of an intercept term and uses the inverse of the outcome variance (quadratic of the standard error) as a weight to provide a comprehensive estimate of the impact of the plasma homocysteine on the incidence of IA. Ensuring these SNPs are not pleiotropic when using the IVW method is important, otherwise, the results will be highly biased. The MR-Egger method can provide causal estimates that are unaffected by breaches of standard IV assumptions and can detect whether standard IV assumptions are violated (25). The weighted median method combines information from various hereditary variations into a solitary causal gauge, and that gauge is predictable even when half of the IVs are null (26).

To test whether horizontal pleiotropy was present among the included SNPs, we performed MR-Egger regression. To examine for a potentially strong impact of an SNP and whether causal effect estimates were reliable, a leave-one-out analysis was performed. In addition, Cochran's *Q*-statistic was applied to examine whether heterogeneity was present among the selected SNPs. We calculated MR power through a web-based tool (https://shiny.cnsgenomics.com/mRnd/) (27). The statistical power under each odds ratio (OR) value was calculated by combining the proportion of cases with IA GWAS, the variance jointly explained by the instrumental variable single nucleotide polymorphisms (SNPs), and the sample size together (Supplementary Table 1). For the primary analysis using serum homocysteine, a relative difference of 21.2% was detected with 80% power (OR: 1.212/0.795) and an alpha value of 5% (Supplementary Table 1). The MR analyses were performed utilizing the TwoSampleMR package for R (version 4.1.2).

## Results

First, we screened 18 SNPs as IVs (genome-wide statistical significance threshold, *P* < 5 × 10^−8^) from a GWAS of plasma homocysteine levels (22). After the removal of SNPs with LD, 13 SNPs remained as IVs (*P* < 5 × 10–8) (rs7422339, rs12134663, rs957140, rs12921383, and rs2851391 were removed). When homocysteine was analyzed against IAs and aneurysmal subarachnoid hemorrhage, two SNPs (rs838133 and rs548987) were found to be absent in the IA and aneurysmal subarachnoid hemorrhage datasets, and when homocysteine was analyzed with unruptured aneurysms, four SNPs (rs838133, rs548987, rs234709, and rs1801133) were absent in the unruptured aneurysm dataset. None of these SNPs have a proxy SNP. The SNPs we used and their association with IAs are shown in Table 1.

**Table 1:** Characteristics of the single nucleotide polymorphisms used as instrumental variables for plasma homocysteine and their association with intracranial aneurysm.

**rsID**	**Gene**	**plasma Hcy level**			**IA (unruptured and ruptured)**			**Unruptured IA**			**Ruptured IA (aSAH)**		
		**β**	**SE**	***p*-value**	**β**	**SE**	***p*-value**	**β**	**SE**	***p*-value**	**β**	**SE**	***p*-value**
rs9369898	MUT	0.045	0.007	2.17E-10	−0.010	0.023	0.669	0.004	0.040	0.93	−0.016	0.027	0.56
rs7130284	NOX4	−0.124	0.013	1.88E-20	−0.020	0.040	0.609	−0.018	0.070	0.80	−0.017	0.046	0.72
rs4660306	MMACHC	0.044	0.007	2.33E-09	−0.004	0.023	0.868	−0.011	0.041	0.78	0.001	0.028	0.99
rs42648	GTPB10	−0.040	0.007	1.97E-08	−0.002	0.023	0.918	−0.004	0.040	0.91	−0.002	0.027	0.94
rs234709	CBS	−0.072	0.007	3.90E-24	−0.030	0.023	0.190	N/A	N/A	N/A	−0.022	0.026	0.39
rs2275565	MTR	−0.054	0.009	1.96E-10	−0.066	0.026	0.010	−0.058	0.047	0.22	−0.062	0.030	0.04
rs2251468	HNF1A	−0.051	0.007	1.28E-12	−0.019	0.022	0.390	−0.003	0.040	0.95	−0.021	0.025	0.39
rs1801222	CUBN	0.045	0.007	8.43E-10	0.017	0.022	0.431	0.029	0.041	0.48	0.010	0.025	0.70
rs1801133	MTHFR	0.158	0.007	4.34E-104	0.034	0.024	0.165	N/A	N/A	N/A	0.035	0.028	0.21
rs154657	DPEP1	0.096	0.007	1.74E-43	−0.018	0.023	0.434	0.008	0.040	0.83	−0.016	0.027	0.56
rs12780845	CUBN	0.053	0.009	7.80E-10	−0.007	0.024	0.780	0.005	0.042	0.91	−0.015	0.029	0.59

aSAH, aneurysmal subarachnoid hemorrhage; IA, intracranial aneurysm; Hcy, homocysteine.

The MR-Egger regression indicated no horizontal pleiotropy in the analysis of the relationship between homocysteine and aneurysms (*P* = 0. 622 for IA, *P* = 0. 491 for aSAH, *P* = 0. 975 for UIA). Furthermore, there were no weak instrumental variables (*F-*statistic: 100.340 for IA and aSAH, and 47.203 for UIA [all >10]). The Chochran's *Q*-statistics showed no significant heterogeneity (*P* = 0.849 for IA, *P* = 0.943 for aSAH, *P* = 0.998 for UIA). The limited number of SNPs included prevented examination of horizontal pleiotropy and heterogeneity.

**Figure 1:**
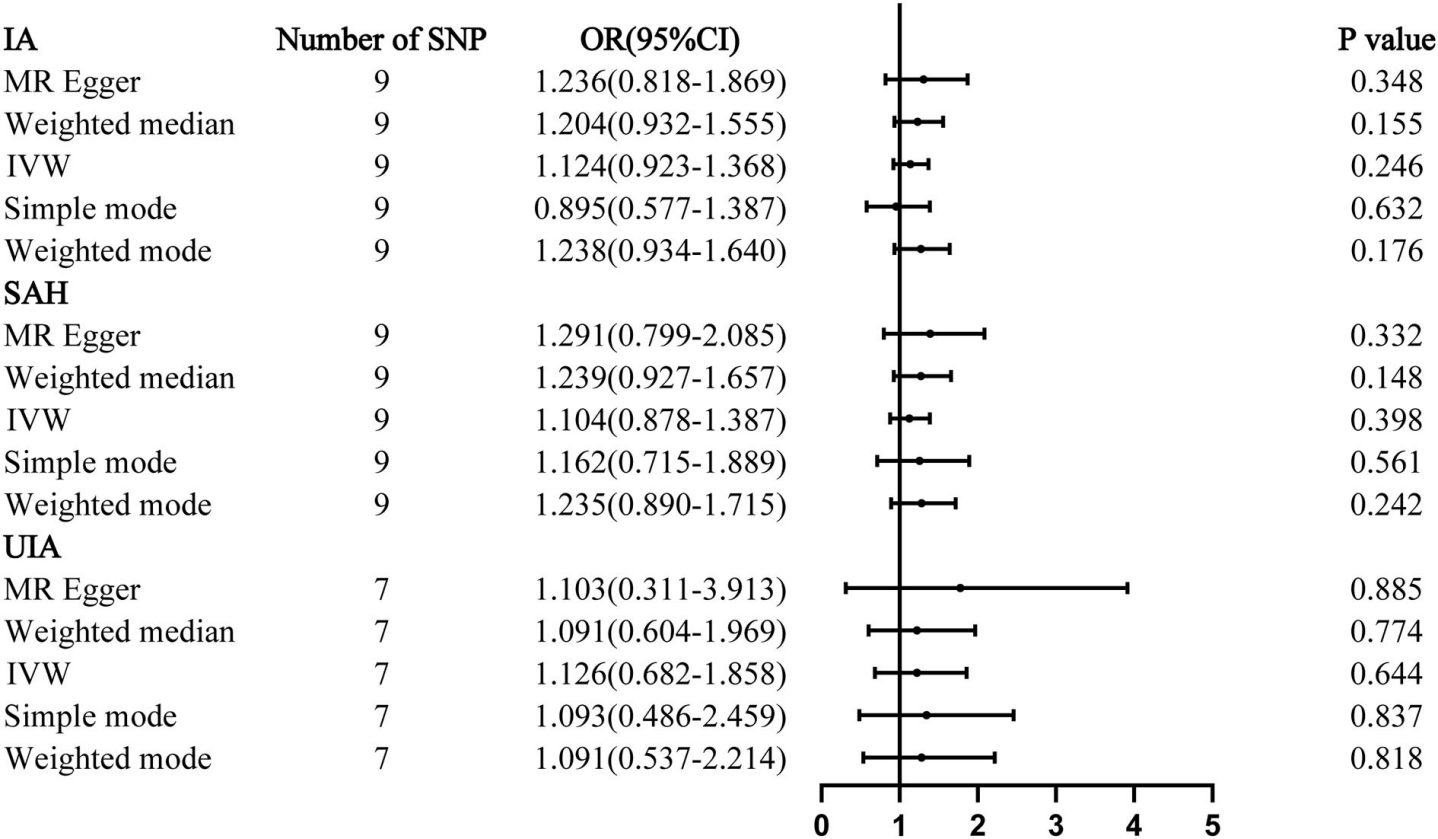
Mendelian randomization analyses of plasma homocysteine levels and the risk of IA. CI, confidence interval; IVW, inverse variance weighted; OR, odds ratio; SAH, subarachnoid hemorrhage; SNP, single-nucleotide polymorphism; UIA, unruptured intracranial aneurysm.

The results of all MR analyses showed no association between IAs and homocysteine, with all *P* > 0.05 (Figure 1). The results of the IVW analysis for aSAH [OR: 1.104; 95% confidence interval (CI): 0.878–1.387, *P* = 0.398], IA (OR: 1.124; 95% CI: 0.923–1.368, *P* = 0.246), and UIA (OR: 1.126; 95% CI: 0.682–1.858, *P* = 0.644) showed no association between IAs and homocysteine.

## Discussion

This MR study provides evidence that IAs are not associated with homocysteine in Europeans. To our knowledge, this is the first MR study on the association between plasma homocysteine levels and IAs.

Based on the data from the Global Burden of Disease Study 2019 (https://www.healthdata.org), stroke is the second leading cause of disability and mortality worldwide (28). Hyperhomocysteinemia has long been recognized as an independent risk factor for stroke (29). Hyperhomocysteinemia is common in the Chinese population (30). Hyperhomocysteinemia can lead to elevated inflammatory factors in blood vessels, damage to the vascular endothelium, and proliferation of vascular smooth muscle cells (31, 32). High homocysteine has been reported to promote atherosclerosis and increase the risk of ischemic strokes (11, 33, 34). Because mechanisms such as inflammation are involved in the formation and rupture of IAs, pathological changes caused by homocysteine may contribute to their formation and rupture. Xu et al. found accelerated IA formation in rats with methionine diet-induced hyperhomocysteinemia (16). Another study showed that methionine-induced hyperhomocysteinemia from excessive methionine intake promotes aneurysmal rupture in orchiectomized rats (35). However, such studies are lacking in humans, and therefore, the relationship between homocysteine and IAs has remained unknown. While some observational studies have reported that IAs are associated with hyperhomocysteinemia in the Chinese population, there is no evidence of a causal link (14, 15). In a Brazilian case-control study, IAs were reported to occur independently of hyperhomocysteinemia, and another study reported that hyperhomocysteinemia is not associated with abdominal aortic aneurysms (17, 36). Thus, the association between IAs and homocysteine remains questionable.

Elevated levels of serum homocysteine mainly cause a decrease in the antithrombotic effect of the vessel wall, increasing the risk of stroke (37). In contrast, aneurysm formation and rupture are mainly considered to be related to damage to the vessel wall and the release of inflammatory factors, and may not be related to the level of homocysteine. Serum levels of homocysteine can be elevated by nutritional deficiencies of folic acid, vitamin B6, and vitamin B12 in the diet. Dietary effects have not been considered in most studies of intracranial aneurysms and homocysteine. Elevated homocysteine levels may also be the result of a ruptured aneurysm; therefore, large prospective studies are still needed to confirm the relationship between aneurysms and homocysteine.

The fundamental benefit of this MR analysis is that estimates of the causal effect of MR were not affected by confounding factors or reverse causal associations found in traditional epidemiological studies. Therefore, compared with observational studies, our current findings may be more reliable. Yet, several limitations remain. First, Genotypic variants in enzymes associated with blood homocysteine levels increase the risk of unprovoked pulmonary embolism (38). Due to the differences in genetic characteristics among different populations, our results may only apply to European populations because all participants in the GWAS were of European origin. Second, not all SNPs were examined, as some were removed because of LD (and no proxy SNPs were found), which may have impacted the results.

At the genetic level, the present MR study suggests that there is no causal relationship between hyperhomocysteinemia and IA or IA rupture. However, further studies are needed to more comprehensively assess the relationship between homocysteine and IAs.

## Data availability statement

The original contributions presented in the study are included in the article/Supplementary material, further inquiries can be directed to the corresponding authors.

## Ethics statement

Ethical review and approval was not required for the study on human participants in accordance with the local legislation and institutional requirements. Written informed consent from the patients/participants or patients/participants' legal guardian/next of kin was not required to participate in this study in accordance with the national legislation and the institutional requirements.

## Author contributions

CM and HL conceived the study. LM, GZ, and YS performed the analyses and manuscript writing. HC and ZL were involved in the acquisition of data. WZ and XX were involved in the interpretation of data. All authors contributed to the article and approved the submitted version.

## Funding

This work was supported by a grant from the National Natural Science Foundation of China (No. 81901258) and the Natural Science Foundation of Jiangsu Province (No. H2017022).

## Conflict of interest

The authors declare that the research was conducted in the absence of any commercial or financial relationships that could be construed as a potential conflict of interest.

## Publisher's note

All claims expressed in this article are solely those of the authors and do not necessarily represent those of their affiliated organizations, or those of the publisher, the editors and the reviewers. Any product that may be evaluated in this article, or claim that may be made by its manufacturer, is not guaranteed or endorsed by the publisher.

## References

[1] Macdonald RL Schweizer TA. Spontaneous subarachnoid haemorrhage. Lancet Lond Engl. (2017) 389:655–66. 10.1016/S0140-6736(16)30668-7 27637674

[2] Vlak MH Algra A Brandenburg R Rinkel GJ. Prevalence of unruptured intracranial aneurysms, with emphasis on sex, age, comorbidity, country, and time period: a systematic review and meta-analysis. Lancet Neurol. (2011) 10:626–36. 10.1016/S1474-4422(11)70109-0 21641282

[3] Chen Y Wright N Guo Y Turnbull I Kartsonaki C Yang L. Mortality and recurrent vascular events after first incident stroke: a 9-year community-based study of 0·5 million Chinese adults. Lancet Glob Health. (2020) 8:e580–90. 10.1016/S2214-109X(20)30069-3 32199124 PMC7090905

[4] Nieuwkamp DJ Setz LE Algra A Linn FHH de Rooij NK Rinkel GJE. Changes in case fatality of aneurysmal subarachnoid hemorrhage over time, according to age, sex, and region: a meta-analysis. Lancet Neurol. (2009) 8:635–42. 10.1016/S1474-4422(09)70126-7 19501022

[5] Piao X Wu G Yang P Shen J De A Wu J. Association between homocysteine and cerebral small vessel disease: a meta-analysis. J Stroke Cerebrovasc Dis. (2018) 27:2423–30. 10.1016/j.jstrokecerebrovasdis.2018.04.035 29801814

[6] Kloppenborg RP Geerlings MI Visseren FL Mali WPTM Vermeulen M van der Graaf Y. Homocysteine and progression of generalized small-vessel disease: the SMART-MR Study. Neurology. (2014) 82:777–83. 10.1212/WNL.0000000000000168 24477110

[7] Jeon S-B Kang D-W Kim JS Kwon SU. Homocysteine, small-vessel disease, and atherosclerosis: an MRI study of 825 stroke patients. Neurology. (2014) 83:695–701. 10.1212/WNL.0000000000000720 25031284

[8] Stehouwer CD Weijenberg MP van den Berg M Jakobs C Feskens EJ Kromhout D. Serum homocysteine and risk of coronary heart disease and cerebrovascular disease in elderly men: a 10-year follow-up. Arterioscler Thromb Vasc Biol. (1998) 18:1895–901. 10.1161/01.ATV.18.12.1895 9848881

[9] Ke XD Foucault-Bertaud A Genovesio C Dignat-George F Lamy E Charpiot P. Homocysteine modulates the proteolytic potential of human arterial smooth muscle cells through a reactive oxygen species dependent mechanism. Mol Cell Biochem. (2010) 335:203–10. 10.1007/s11010-009-0270-7 19787299

[10] Tawfik A Elsherbiny NM Zaidi Y Rajpurohit P. Homocysteine and age-related central nervous system diseases: role of inflammation. Int J Mol Sci. (2021) 22:6259. 10.3390/ijms22126259 34200792 PMC8230490

[11] McCully KS. Homocysteine metabolism, atherosclerosis, and diseases of aging. Compr Physiol. (2015) 6:471–505. 10.1002/cphy.c150021 26756640

[12] Frösen J Tulamo R Heikura T Sammalkorpi S Niemelä M Hernesniemi J. Lipid accumulation, lipid oxidation, and low plasma levels of acquired antibodies against oxidized lipids associate with degeneration and rupture of the intracranial aneurysm wall. Acta Neuropathol Commun. (2013) 1:71. 10.1186/2051-5960-1-71 24252658 PMC3893371

[13] Turjman AS Turjman F Edelman ER. Role of fluid dynamics and inflammation in intracranial aneurysm formation. Circulation. (2014) 129:373–82. 10.1161/CIRCULATIONAHA.113.001444 24446407 PMC4371596

[14] Wang Q Zhang J Zhao K Xu B. Hyperhomocysteinemia is an independent risk factor for intracranial aneurysms: a case-control study in a Chinese Han population. Neurosurg Rev. (2020) 43:1127–34. 10.1007/s10143-019-01138-9 31256274

[15] Ren J-R Ren S-H Ning B Wu J Cao Y Ding X-M. Hyperhomocysteinemia as a risk factor for saccular intracranial aneurysm: a cohort study in a Chinese Han population. J Stroke Cerebrovasc Dis. (2017) 26:2720–6. 10.1016/j.jstrokecerebrovasdis.2017.01.001 28943219

[16] Xu Y Tian Y Wei H-J Dong J-F Zhang J-N. Methionine diet-induced hyperhomocysteinemia accelerates cerebral aneurysm formation in rats. Neurosci Lett. (2011) 494:139–44. 10.1016/j.neulet.2011.02.076 21382440

[17] Rosi J Morais BA Pecorino LS Oliveira AR Solla DJF Teixeira MJ. Hyperhomocysteinemia as a risk factor for intracranial aneurysms: a case-control study. World Neurosurg. (2018) 119:e272–5. 10.1016/j.wneu.2018.07.132 30053565

[18] Davey Smith G Hemani G. Mendelian randomization: genetic anchors for causal inference in epidemiological studies. Hum Mol Genet. (2014) 23:R89–98. 10.1093/hmg/ddu328 25064373 PMC4170722

[19] Tan J-S Liu N Guo T-T Hu S Hua L Qian Q. Genetic predispositions between COVID-19 and three cardio-cerebrovascular diseases. Front Genet. (2022) 13:743905. 10.3389/fgene.2022.743905 35368685 PMC8966609

[20] Tan J-S Liu N-N Guo T-T Hu S Hua L. Genetically predicted obesity and risk of deep vein thrombosis. Thromb Res. (2021) 207:16–24. 10.1016/j.thromres.2021.08.026 34507265

[21] Liu N Tan J-S Liu L Wang Y Hua L Qian Q. Genetic predisposition between COVID-19 and four mental illnesses: a bidirectional, two-sample Mendelian randomization study. Front Psychiatry. (2021) 12:746276. 10.3389/fpsyt.2021.746276 34744839 PMC8564104

[22] van Meurs JB Pare G Schwartz SM Hazra A Tanaka T Vermeulen SH. Common genetic loci influencing plasma homocysteine concentrations and their effect on risk of coronary artery disease12345. Am J Clin Nutr. (2013) 98:668–76. 10.3945/ajcn.112.044545 23824729 PMC4321227

[23] Bakker MK van der Spek RAA van Rheenen W Morel S Bourcier R Hostettler IC. Genome-wide association study of intracranial aneurysms identifies 17 risk loci and genetic overlap with clinical risk factors. Nat Genet. (2020) 52:1303–13. 10.1038/s41588-020-00725-7 33199917 PMC7116530

[24] Didelez V Sheehan N. Mendelian randomization as an instrumental variable approach to causal inference. Stat Methods Med Res. (2007) 16:309–30. 10.1177/0962280206077743 17715159

[25] Bowden J Davey Smith G Burgess S. Mendelian randomization with invalid instruments: effect estimation and bias detection through Egger regression. Int J Epidemiol. (2015) 44:512–25. 10.1093/ije/dyv080 26050253 PMC4469799

[26] Bowden J Davey Smith G Haycock PC Burgess S. Consistent estimation in Mendelian randomization with some invalid instruments using a weighted median estimator. Genet Epidemiol. (2016) 40:304–14. 10.1002/gepi.21965 27061298 PMC4849733

[27] Brion M-JA Shakhbazov K Visscher PM. Calculating statistical power in Mendelian randomization studies. Int J Epidemiol. (2013) 42:1497–501. 10.1093/ije/dyt179 24159078 PMC3807619

[28] GBD 2016 Stroke Collaborators. Global, regional, and national burden of stroke, 1990-2016: a systematic analysis for the Global Burden of Disease Study 2016. Lancet Neurol. (2019) 18:439–58. 10.1016/S1474-4422(19)30034-1 30871944 PMC6494974

[29] Hankey GJ Eikelboom JW. Homocysteine and stroke. Lancet Lond Engl. (2005) 365:194–6. 10.1016/S0140-6736(05)70126-4 15652586

[30] Tu W Yan F Chao B Ji X Wang L. Status of hyperhomocysteinemia in China: results from the China stroke high-risk population screening program, 2018. Front Med. (2021) 15:903–12. 10.1007/s11684-021-0871-4 34893949

[31] Zou T Yang W Hou Z Yang J. Homocysteine enhances cell proliferation in vascular smooth muscle cells: role of p38 MAPK and p47phox. Acta Biochim Biophys Sin. (2010) 42:908–15. 10.1093/abbs/gmq102 21068125

[32] Balint B Jepchumba VK Guéant J-L Guéant-Rodriguez R-M. Mechanisms of homocysteine-induced damage to the endothelial, medial and adventitial layers of the arterial wall. Biochimie. (2020) 173:100–6. 10.1016/j.biochi.2020.02.012 32105811

[33] Lehotský J Tothová B Kovalská M Dobrota D Benová A Kalenská D. Role of homocysteine in the ischemic stroke and development of ischemic tolerance. Front Neurosci. (2016) 10:538. 10.3389/fnins.2016.00538 27932944 PMC5120102

[34] Xiong J Ma F Ding N Xu L Ma S Yang A. miR-195-3p alleviates homocysteine-mediated atherosclerosis by targeting IL-31 through its epigenetics modifications. Aging Cell. (2021) 20:e13485. 10.1111/acel.13485 34592792 PMC8520716

[35] Korai M Kitazato KT Tada Y Miyamoto T Shimada K Matsushita N. Hyperhomocysteinemia induced by excessive methionine intake promotes rupture of cerebral aneurysms in ovariectomized rats. J Neuroinflammation. (2016) 13:165. 10.1186/s12974-016-0634-3 27349749 PMC4924228

[36] Vats S Sundquist K Wang X Zarrouk M Ågren-Witteschus S Sundquist J. Associations of global DNA methylation and homocysteine levels with abdominal aortic aneurysm: a cohort study from a population-based screening program in Sweden. Int J Cardiol. (2020) 321:137–42. 10.1016/j.ijcard.2020.06.022 32593727

[37] Carrizzo A Iside C Nebbioso A Carafa V Damato A Sciarretta S. SIRT1 pharmacological activation rescues vascular dysfunction and prevents thrombosis in MTHFR deficiency. Cell Mol Life Sci. (2022) 79:410. 10.1007/s00018-022-04429-5 35821533 PMC9276577

[38] Tan J-S Yan X-X Wu Y Gao X Xu X-Q Jiang X. Rare variants in MTHFR predispose to occurrence and recurrence of pulmonary embolism. Int J Cardiol. (2021) 331:236–42. 10.1016/j.ijcard.2021.01.073 33571559

