# Supplementary material for: Hyperhomocysteinemia and intracranial aneurysm: A mendelian randomization study
Source: Front Neurol. 2022 Sep 28;13:948989. doi: 10.3389/fneur.2022.948989 (PMC9554923; doi:10.3389/fneur.2022.948989)
Supplement: Supplementary file 1 [file Table_1.pdf]

Table S1 Power to detect a relative difference (in %) in IA risk for a 1-unit difference in log-transformed serum homocysteine with an alpha of 5%

	relative difference in %																
	5%	10%	15%	20%	21.20%	25%	25.30%	30%	35%	40%	45%	50.00%	55.00%	55.20%	60%	65%	70%
IA	0.10	0.26	0.51	0.75	0.80	0.91	/	0.97	1.00	1.00	1.00	1.00	1.00	/	1.00	1.00	1.00
SAH	0.09	0.20	0.39	0.60	/	0.79	0.80	0.91	0.97	0.99	1.00	1.00	1.00	/	1.00	1.00	1.00
uIA	0.06	0.08	0.12	0.17	/	0.25	/	0.33	0.43	0.53	0.62	0.71	0.79	0.80	0.86	0.91	0.94

IA, intracranial aneurysm; SAH, subarachnoid hemorrhage; uIA, unruptured intracranial aneurysm.
